# Multidisciplinary development of guidelines for ketamine treatment for treatment-resistant major depression disorder for use by adult specialist mental health services in New Zealand

**DOI:** 10.1192/bjo.2023.577

**Published:** 2023-10-13

**Authors:** Ben Beaglehole, Paul Glue, Mike Clarke, Richard Porter

**Affiliations:** Department of Psychological Medicine, University of Otago, New Zealand; Specialist Mental Health Services, Te Whatu Ora – Health New Zealand Waitaha Canterbury, New Zealand

**Keywords:** Ketamine, evidence base, guidelines, protocol, oral ketamine

## Abstract

**Background:**

The evidence base for racemic ketamine treatment for treatment-resistant major depressive disorder (TRD) continues to expand, but there are major challenges translating this evidence base into routine clinical care.

**Aim:**

To prepare guidelines for ketamine treatment of TRD that are suitable for routine use by publicly funded specialist mental health services.

**Method:**

We consulted with senior leadership, clinical pharmacy, psychiatrists, nursing, service users and Māori mental health workers on issues relating to ketamine treatment. We prepared treatment guidelines taking the evidence base for ketamine treatment and the consultation into account.

**Results:**

Ketamine treatment guidance is reported. This offers two treatment pathways, including a test of ketamine responsiveness with intramuscular ketamine and the dominant use of oral ketamine for a 3-month course to maximise the opportunity for the short-term benefits of ketamine to accumulate.

**Conclusions:**

We have responded to the challenges of translating the evidence base for ketamine treatment into a form suitable for routine care.

Treatment-resistant major depressive disorder (TRD) is burdensome for individuals, families and society.^1^ Existing treatments for TRD have limitations, including side-effects and limited effectiveness of psychotherapy and medication treatments.^2^ It is therefore desirable that newly established treatments become available for patients in a timely manner. The N-methyl-D-aspartate receptor antagonist ketamine has emerged as a new treatment for major depressive disorder (MDD). Although the efficacy of ketamine treatment for TRD in the short term is now well established,^3^ there are a number of challenges for the translation of ketamine treatment to routine care. These include the following: high rates of relapse that occur following cessation of regular dosing,^4,5^ marked dissociative effects during parenteral dosing,^3^ concern about the potential for dependence and misuse,^6^ and concern about other side-effects such as interstitial cystitis^7,8^ and memory impairment.^9,10^

An obvious challenge for mental health services is the matching of clinical resource to clinical need, and the rollout of new treatments in the most equitable way. In New Zealand, esketamine (the S-enantiomer of ketamine) is available for TRD in combination with a conventional antidepressant, and is delivered via nasal spray. Esketamine is not funded by Pharmac (the government agency that decides which medications are funded in New Zealand), and is very costly for consumers. Royal Australian and New Zealand College of Psychiatry (RANZCP) clinical guidelines also report caveats that esketamine has not been compared directly with ketamine, the majority of esketamine data stems from industry-sponsored trials and longer-term outcomes with this formulation are still a matter for debate.^11^ Racemic ketamine (combined esketamine and arketamine) does not have regulatory approval for the treatment of MDD in New Zealand. Despite this, limited off-label ketamine use occurs in clinical and research settings, and demand from patients and clinicians for ketamine treatment is increasing. However, the routine use of ketamine in publicly funded specialist mental health services (SMHS) is largely absent. In large part, this relates to the challenges outlined earlier, accompanied by lack of expertise in ketamine treatment in clinicians.

## Response to the challenges outlined above

We created treatment guidelines for ketamine use in publicly funded mental health services in response to the challenge of translating ketamine use to clinical practise. A primary goal was to provide a pathway for consumers with TRD to receive ketamine treatment. A secondary aim was to provide a framework for other services to follow and establish ketamine treatment in their treatment settings. This paper describes the process undertaken in creating the ketamine treatment guidelines. At the time of submitting this paper for review, the protocol remained in draft form. Although consultation was complete and there was general agreement with internal stakeholders on the content of the protocol, systemic changes affecting New Zealand health services nationally (the transition from District Health Boards to a single national healthcare entity, Te Whatu Ora) meant that new healthcare initiatives were not being progressed locally. Despite remaining in draft form, we believe there is merit in reporting the process undertaken and the protocol, to allow for broader dissemination and review.

## Method

The authors assert that all procedures contributing to this work comply with the ethical standards of the relevant national and institutional committees on human experimentation and with the Helsinki Declaration of 1975, as revised in 2008. There were no procedures involving human patients.

### Setting

Our SMHS provides government-funded health services to a predominantly urban catchment area. It is responsible for the health of approximately 600 000 people (comprising approximately 81.1% New Zealand European, 9.5% Māori, 12.4% Asian and 3.3% Pacifika). Comprehensive mental health services are available, including specialist child and adolescent, adult and old-age services, in in-patient and out-patient settings. Private psychiatry is minimal and without in-patient facilities.

### Leadership

Before commencing the development of the ketamine treatment guidelines, we consulted with the Chief of Psychiatry at the SMHS, to ensure there would be service support for ketamine use. Following the endorsement by the Chief of Psychiatry, the Clinical Director of one of the adult community psychiatric teams was nominated as a key figure and member of the steering group.

### Process and consultation

The first draft of the protocol was largely written by author B.B., drawing upon ketamine protocols used in research settings with P.G. This is unpublished work to date that utilises intramuscular and oral ketamine treatment. Following completion of the first draft an iterative process was undertaken. This involved circulating draft protocols before meetings with consultation groups. These groups included leadership and community psychiatrists, the SMHS pharmacy department, the electroconvulsive therapy (ECT) service, nursing representatives, Te Korowai Atawhai (the local Māori mental health service) and patient representatives. After each meeting, feedback received was used to revise and improve the protocol. We anticipate that there may be further versions of the protocol in response to the audit and further developments in the evidence base.

### Steering group

When finalised, we anticipate a steering group to oversee ketamine use until it becomes an established intervention. Representatives from SMHS leadership (Clinical Director of community team), academic psychiatry, pharmacy, the ECT service, nursing representatives, patient representatives and Te Korowai Atawhai (the Māori mental health service) will constitute the steering group. We anticipate 3-monthly meetings to review ketamine audits, address issues as they arise and oversee ketamine treatment. Potential issues that may arise for the steering group include training issues in intramuscular administration, resourcing for intramuscular administration, patient selection and questions about management of relapse following treatment.

### Training

Author B.B. presented the protocol to community psychiatrists and provides consultation and clinical back-up to SMHS clinicians who have commenced ketamine treatment using the draft protocol. Author P.G. provides additional ketamine experience, expertise and support. Author M.C. completed training in ketamine treatment during a recent sabbatical. It is intended that psychiatrists will observe three intramuscular treatments to ensure familiarity with the dissociative ketamine experience before overseeing intramuscular ketamine administration for future patients. Oral ketamine can be provided using the protocol for guidance, given the limited dissociation that occurs with oral dosing. National workshops for psychiatrists in other regions seeking to familiarise themselves with ketamine treatment and the setting up of ketamine clinics are planned.

### Internal benchmarking with RANZCP clinical memorandum on use of ketamine in psychiatric practice

The RANZCP released a clinical memorandum for psychiatrists considering ketamine use in clinical practice.^12^ This guideline contains key messages. In devising this guideline, we aimed to be aligned with these messages. After completion of the guideline, we undertook an internal benchmarking process. This entailed comparing the key messages of the RANZCP clinical memorandum with our guideline. There was broad agreement with all key messages apart from partial agreement with the requirement that a psychiatrist with appropriate expertise in ketamine treatment should prescribe ketamine and take responsibility for its use. Appropriate expertise is not well defined. In our view, the predominant use of oral ketamine and other layers of oversight provided by our guideline ensure sound ketamine practice.

### Audit

Our clinical research unit agreed to collate data on ketamine treatments. Sociodemographic details and specifics relating to ketamine treatment, including rating scale scores (e.g. for the Depression, Anxiety and Stress Scale; DASS-21^13^) and any adverse effects, will be reported to the 3-monthly steering group meeting. The data will be collated by the case manager. We considered using the Ketamine Side Effect Tool (KSET)^14^ to monitor side-effects, but chose not to do so because of the complexity of this tool. Supplementary File 1 available at https://doi.org/10.1192/bjo.2023.577 is our suggested form to document ketamine treatment and side-effects.

## Results

The draft ketamine treatment guidelines for adults aged 18–65 years are provided in Supplementary File 2. Table 1 provides key elements of the guidelines that relate to treatment provision for ease of reader review. Table 2 contains the patient information leaflet to assist informed decision-making. Table 3 provides the consent for ketamine treatment form to be used before treatment commencement for each patient.

**Table 1:** Key elements of the ketamine clinic treatment guidelines

**Primary indication**
The primary patient population will be adult patients with treatment-resistant depression (TRD). Given the challenges associated with ketamine treatment, we propose restricting ketamine treatment to those with TRD of a severity to require treatment by specialist mental health services. Proposed patients will therefore be receiving treatment for TRD from adult community psychiatric services and be allocated a case manager and overseeing psychiatrist. We expect patients with TRD to have trialled at least two antidepressant treatments (of adequate dose and duration) and evidence-based psychotherapy for major depressive disorder (if available).
**Exclusion criteria**
Inability to consent for treatment Significant comorbid personality disorder Active substance use disorder Primary psychotic disorder Dementia Significant bladder pathology Allergy to ketamine Significant hypertension/cardiovascular disease (for the rapid test of ketamine response pathway only) Other significant acute or chronic medical comorbidity Failed course of ketamine in the past 12 months
**Ketamine treatment**
We include two ketamine treatment options. Clinicians and patients to select one of the following: Rapid test of ketamine response pathway If determining whether patient is responsive to ketamine is urgent, patients can be given 1 mg/kg intramuscular ketamine at the clinic of the local psychiatric hospital (where medical monitoring and support is available). If this pathway is chosen, the case manager and psychiatrist will both attend for ketamine dosing and remain until the acute dissociative reaction has subsided. Close supports/*whānau* may also attend. If the psychiatrist has observed fewer than four intramuscular ketamine administrations, a medical practitioner experienced in ketamine treatment is required to support treatment administration. Pre-medication will include 4 mg ondansetron orally at 30 min pre-dosing. Ketamine monitoring will include baseline blood pressure, oxygen saturations and heart rate. Ketamine administration not to proceed if blood pressure is >140/90 at baseline or heart rate is >100/min. Blood pressure and heart rate should be monitored at 15-min intervals until returned to baseline. Patients should expect to be at the clinical services unit for 3 h, including 2 h post-dose. Patients need to be oriented, alert, mobile and with stable observations before departure. Self-report Depression, Anxiety and Stress Scale (DASS-21) to be completed at baseline and 48 h post-treatment, to check if ketamine responsive and whether to continue with the oral ketamine pathway. Repeated intramuscular injections are outside of the pathway. If the patient is ketamine responsive, they will have the option of continuing with the standard oral ketamine pathway outlined next. Standard oral ketamine pathway Overseeing psychiatrist to prescribe ketamine according to protocol and discuss with a medical practitioner experienced in ketamine treatment if needed. Case manager and/or team nurse to administer oral ketamine in an appropriate setting (home or clinic) and provide one-to-one support during first dose. Supports/*whānau* welcome to attend. Standard racemic ketamine for injection to be used mixed with 100 ml orange juice and sipped over 30–60 min. Oral dosing and sipping for over 30 min will prevent significant dissociation and sympathetic response, by minimising peak drug concentrations. Initial dose 1 mg/kg. According to DASS-21 and clinical response, ketamine can be increased to 1.5 mg/kg and then 2 mg/kg if response is suboptimal. Dose frequency is once or twice weekly depending on duration of response. Exceptionally this could be three times per week, but only for patients where there is a clear improvement post-dosing, and a rapid return of depressive symptoms. The course of ketamine is up to 12 weeks. This duration was chosen to provide sufficient opportunity for a robust, sustained clinical response with opportunity for behavioural change to increase the likelihood of a more enduring improvement in well-being.
**Informed consent**
Informed consent is required to proceed with ketamine treatment. This process will be completed by the treating psychiatrist. The decision-making process will be documented using a written consent form to ensure the following are discussed: That ketamine is being used off label The risks and benefits of ketamine treatment, and alternative treatments The dissociative reaction associated with IM ketamine is explained if this pathway is chosen The short-term nature of ketamine response, and the high risk of relapse following the end of the course of treatment The potential for dependence and misuse Ketamine bladder/interstitial cystitis among high-dose and high-frequency recreational ketamine users Cognitive/memory impairment among high-dose and high-frequency recreational ketamine users The monitoring requirements for participation in the ketamine clinic
**Monitoring**
Mood: Patients will be monitored clinically by their case managers on at least a weekly basis during ketamine treatment. Ketamine treatment monitoring will be supplemented by the DASS-21^a^ at baseline and at least weekly intervals during ketamine treatment. A further DASS-21 will be completed 6 weeks after treatment end. The baseline and weekly DASS-21 scores will be collected by the patient's case manager, and prompt regular discussion with the psychiatrist about the clinical status of the patient, including any risk issues and the adequacy of dose. Psychiatrist review should occur the week following the intramuscular ketamine dose (if used) or 2 weeks after ketamine initiation, and then as needed. Cognition: In most cases this will be monitored simply by enquiring with the patient and family/*whānau* if available. More formal testing will be considered if there are complaints of cognitive difficulties. Screening for urinary tract symptoms will be undertaken pre-ketamine treatment and monitored monthly during the course of treatment. Suggested screening questions are provided in Supplementary File 1.

**Table 2:** Suggested patient information leaflet: ketamine treatment for treatment-resistant depression

**What is treatment-resistant depression (TRD)?**
Depression is the most common mental illness in the community. Depression is characterised by feeling low in mood and not being able to enjoy life. Depression is usually accompanied by over- or under-sleeping, appetite changes, energy changes and impaired concentration. Thinking usually becomes negative and may include suicidal thoughts. TRD is diagnosed when depression persists despite trying antidepressants and psychotherapy (talk therapy).
**What is ketamine?**
Ketamine is a medication used in the management of severe pain and sometimes anaesthesia. For the past decade or more, clinicians have studied ketamine for the treatment of mental illnesses.
**Ketamine treatment for TRD**
Ketamine is an effective short-term treatment for TRD, although not everybody tolerates ketamine or responds to treatment. The majority of studies have investigated ketamine injections, but oral ketamine (ketamine mixed with orange juice and sipped over 30–60 min) has also been used and appears beneficial. Ketamine use is off-label in New Zealand, although Esketamine (closely related to ketamine) is registered for TRD. Esketamine is not funded and is therefore expensive for consumers.
**Side-effects of ketamine**
Ketamine injections are associated with a severe ‘dissociative’ experience. Patients feel disconnected from their usual senses and time for approximately 45 min. They may become drowsy or immersed in past memories. Nausea is common, so we provide an anti-nausea medication (ondansetron) before the injection. Oral ketamine is usually better tolerated with minimal dissociation or nausea. Ketamine causes short-term increases in blood pressure and heart rate that subside as the effects wear off. Ketamine is a recreational drug used by some people. It can be associated with dependence or addiction. Frequent, high-dose use of ketamine by drug users is associated with bladder symptoms (difficulty passing urine) and memory impairment (forgetfulness), but these concerns do not seem significant when ketamine is used for psychiatric reasons.
**Important things to consider**
We offer a single intramuscular injection of ketamine as a rapid way of determining whether you are likely to respond to oral ketamine. The dissociative effects of ketamine can be scary, although many people manage the experience without much difficulty. If you are responsive to ketamine, you will be offered a course of oral ketamine to follow. If you take regular medications, these will not change and you are advised to continue taking them as prescribed throughout ketamine treatment. If you do not wish to have the injection you can still receive the course of oral ketamine. We anticipate this will be more suitable for most people as it avoids the injection and the severe dissociative experience. There may be some situations, such as severe suicidal thoughts, where your psychiatrist may recommend starting with the injection as a better option. Ketamine is only associated with short-term benefits. Typically, mood feels better for several days before deteriorating again. Repeated doses offer the possibility of more sustained improvement during the course of treatment. The risk of depression relapse is still high after single or repeated doses. We offer ketamine treatment as a 12-week course. We believe this time frame provides a good opportunity for better control of symptoms. We believe the course of treatment also offers you the chance to make changes that may prevent relapse after ketamine stops. It is important that you discuss ketamine treatment with your family/*whānau* and your treating team before deciding if you want to proceed with ketamine treatment.

**Table 3:** Suggested consent form for off-label ketamine treatment of depression

This form is to be completed following a discussion about ketamine treatment and its alternatives with the consultant psychiatrist overseeing care. It is important to take the time to make a good decision and discuss ketamine treatment with family/*whānau* and other supports. I, …………………………………………………………………………………….., consent to receiving treatment with one of the following (please delete the non-applicable option):
Rapid test of ketamine response option (intramuscular injection of ketamine. If ketamine responsive, a course of oral ketamine once or twice weekly for a maximum of 12 consecutive weeks will be offered) Standard oral ketamine pathway (oral ketamine once or twice weekly for a maximum of 12 consecutive weeks).
In giving consent, I acknowledge that I have discussed the pros and cons of ketamine treatment, including alternative options, with my psychiatrist. This discussion has included the following:
The intense dissociative reaction (spacing out) experience with intramuscular ketamine. Dissociative side-effects of intramuscular ketamine include time speeding or slowing, colours changing in intensity, reliving old memories and altered sensations (delete if not applicable) The risk of high blood pressure with intramuscular ketamine (delete if not applicable) Oral ketamine is associated with temporary mild effects including disinhibition and euphoria (extreme happiness) The short-term clinical response to ketamine The risk of relapse following a course of ketamine treatment The risk of bladder symptoms following ketamine treatment (this is largely an issue with high-dose frequent recreational users) The risk of memory problems with ketamine treatment (this is largely an issue with high-dose frequent recreational users) The risks of recreational/misuse of ketamine
Signed: ………………………………………………………………………………………………..date…………………………………………….. Psychiatrist: Name……………………………………………………..Signature…………………………………………………..date………………… Psychiatrist: Name……………………………………………………..Signature…………………………………………………..date…………………

## Discussion

This paper describes the process undertaken to develop ketamine treatment guidelines for use in a publicly funded mental healthcare setting. The ketamine clinic guidelines that resulted are provided to inform wider ketamine use by psychiatrists in other settings. In undertaking this work, we respond to the increasing evidence base for ketamine treatment of TRD and the gap that exists in translating evidence from clinical trials into use by clinicians. We believe the guidelines are a pragmatic reflection of the evidence base for ketamine treatment that provide a useful resource for services and clinicians, particularly in jurisdictions where resources for mental health treatment are significantly limited.

A further impetus for this work was the increasing awareness of new treatments for TRD in public domains. We were aware of increasing demand and pressure on individual clinicians to respond to requests for ketamine treatment. In this context, a SMHS-endorsed treatment pathway appeared desirable compared with *ad hoc* individual clinician-led solutions. Therefore, a critical feature in developing this guideline was the early involvement of SMHS leadership, wide consultation and planned ongoing review and audit of ketamine treatment.

The majority of treatment studies evaluating ketamine treatment for TRD use parenteral administration of ketamine, but ketamine is active in TRD irrespective of how it is administered.^3^ The pharmacology of ketamine relating to its antidepressant activity has been linked to several of its metabolites, including norketamine and the hydroxynorketamines.^15,16^ After oral dosing, pharmacokinetic exposure to norketamine and the hydroxynorketamines is considerably more prolonged than exposure to ketamine.^17^ Furthermore, ketamine is still active as an antidepressant even when dosed by routes where bioavailability of parent ketamine is low.^18^ A synthesis of these observations suggests that ketamine may be acting as a prodrug, where its antidepressant activity is substantially attributable to its metabolites. A meta-analysis of ketamine formulations identified that formulations that maximise first-pass metabolism of ketamine and delay time to maximum concentrations were better tolerated (less dissociation) and safer (less blood pressure change) than formulations that lack those characteristics.^19^ Our treatment guidelines allow for intramuscular ketamine as a test of ketamine responsiveness if desired by the clinical team and patient. We chose the dose of 1 mg/kg because of recent clinical trial and ‘real-world’ evidence that 0.5 mg/kg is an ineffective dose for some, and to optimise the likelihood of response.^20,21^ Alternative routes include intravenous and subcutaneous administration. These have been shown to produce similar antidepressant effects in a small ascending dose study, although plasma ketamine levels are higher when given intravenously.^22^ This may prompt the need for dose adjustment if this route is preferred. In the study by Loo et al,^22^ the subcutaneous route of administration was better tolerated than other forms of administration, but we recommended intramuscular administration because of greatest familiarity with this route of administration locally, and because subcutaneous administration is not endorsed by Medsafe, the regulatory body overseeing medication use in New Zealand.

Following the intramuscular test and as an alternative pathway, ketamine treatment is provided orally. We chose this to allow more widespread use of ketamine, but recognise that there may be situations when repeated doses of intramuscular ketamine are preferred (particularly severe suicidality). The evidence base for oral treatment is less extensive than for parenteral administration, but oral ketamine administration is better tolerated, with minimal dissociative symptoms at the time of ingestion.^23,24^ Oral ketamine is also associated with medium-to-large effect size improvements in depression severity after 2–6 weeks of treatment.^23,24^ The decision to recommend oral ketamine is not without controversy because the majority of studies have still been with parenteral administration. The tolerability and ease of administration of oral ketamine was a key factor in our decision to prefer oral treatment. We recognised that delivering parenteral ketamine in busy psychiatric settings was an obstacle to ketamine use in our SMHS, and this is likely to be similar in other publicly funded SMHS. We were also concerned about issues of equity and access. Currently, esketamine and ketamine treatment is available for consumers who are able to seek treatment in private settings at considerable expense. We wished to increase access to other consumers, and the likelihood of parental administration of ketamine or esketamine becoming prevalent in public services seemed low. Also contributing to this decision was the established use of oral ketamine in research settings in Dunedin and Christchurch, New Zealand.

A key challenge to ketamine treatment is the short-term nature of beneficial effects, with high rates of relapse following single and repeated dosing.^4,5^ This finding creates a dilemma for clinicians: determining the best duration of ketamine treatment, including whether to commit to long-term or maintenance treatment. Our guidelines specify that the duration of ketamine treatment should be up to 12 weeks. Twelve weeks was chosen to provide sufficient time for a robust clinical response. We also believe that a 12-week course provides opportunity for behavioural activation and the reinforcement of mood improvements by behavioural change. However, we were reluctant to recommend long-term or maintenance treatment of racemic ketamine because controlled trials supporting this approach are not yet published,^25^ and we were concerned that the clinical resource for ketamine treatment could be overwhelmed. However, regular steering group meetings provide a mechanism for change to the ketamine guidelines if ongoing audit suggests that the time frame for treatment is unfeasible.

The extent and burden of TRD in the community is significant,^1^ but our guidelines limit access to ketamine treatment to those with severe TRD under SMHS care. This was a practical decision taken as the first step toward greater access to ketamine treatment in our local community. In doing so, we prioritise access to ketamine treatment for those with most severe TRD and ensure that treating psychiatrists are at the forefront of ketamine treatment. We did not wish to create a situation where psychiatrists were required to address issues with ketamine treatment provided in other settings that were not endorsed by SMHS. We recognise that this approach may not fully address the community need for ketamine treatment, but believe it is justified given the risks of ketamine treatment and longer-term side-effects.

Although a large part of the ketamine treatment evidence base addresses the management of TRD, there is growing evidence for the use of ketamine treatment for other psychiatric disorders, including anxiety and substance use disorders.^26^ We anticipate that the indication for ketamine treatment will broaden with time, but recommend restricting treatment to TRD initially as a pragmatic response, given the uncertain extent of demand following opening a new service.

There are studies that suggest additional benefits from combining psychotherapy and ketamine treatment for TRD.^27^ This evidence base is expanding, but given that moderate-to-large effect size improvements result from oral ketamine treatment for TRD alone,^23^ specifying that psychotherapy be required did not appear justified. However, we do require patients receiving ketamine to be managed by SMHS. We regard the relationship with a case manager and psychiatrist to be an important adjunct to the ketamine treatment that will help embed behavioural change alongside any improvements in mood.

The RANZCP has released a clinical memorandum to provide information for psychiatrists about the potential utility of ketamine in psychiatric practice.^12^ We internally bench-marked our guidelines against the key messages of this memorandum. Our guidelines were consistent with the key messages, with the exception of the requirement for the treating psychiatrist to have expertise in ketamine treatment. In our view, the use of oral ketamine and the oversight provided by these guidelines, alongside clinical support from psychiatrists with more ketamine treatment expertise, means that psychiatrists treating patients with TRD can initiate and supervise the use of oral ketamine treatment without extensive prior ketamine treatment experience, and will gain this in the process specified by this protocol.

The wide consultation we undertook allowed for refinement of the protocol in a number of areas. For example, the importance of a careful informed consent process balancing the risks and benefits was emphasised during patient and Te Korowai Atawhai consultation. Community psychiatrists were largely supportive, but not universally so, with concern about off-label use and the lack of translational evidence for ketamine. Clinical pharmacy expertise helped refine protocol and advice for patients considering ketamine treatment. Each of the groups consulted were supportive of a protocol to support ketamine use by SMHS.

### Limitations

This protocol is intended to provide a useful ketamine pathway for the treatment of patients with TRD. The definition of TRD used is failure to respond to adequate trials of two antidepressants and evidence-based psychotherapy (if available), and of a sufficient severity to require management by SMHS. The concept of TRD can be challenged on multiple fronts, including unrecognised bipolarity, heterogeneity of presentation and inadequate treatments (as opposed to treatment resistance residing within the patient).^28^ We accept these criticisms of TRD, but suggest that some criteria are desirable for pragmatic reasons. We developed this guideline to provide a useful protocol for general adult services. Consequently, the use of ketamine in subspecialty areas such as child and adolescent psychiatry and maternal and infant health is not addressed, and should prompt the use of clinical pharmacy and psychiatric second opinions if considered.

This ketamine protocol is a local solution responding to the increasing evidence base for ketamine treatment and demand from clinicians and patients for greater availability of ketamine treatment for TRD. It remains in draft form because of an inability to progress the protocol to a finalised status in the context of recent structural changes to the provision of healthcare in New Zealand. Although designed with our local SMHS in mind, we believe that our local service characteristics can be generalised to other publicly funded SMHS, such as in Australia and the National Health Service in the UK. Our guideline specifies the use of oral ketamine despite a larger portion of the literature investigating parenteral ketamine. This was a pragmatic decision, and better-resourced services may choose to prioritise parenteral administration as a first choice despite obvious tolerability and resource advantages to oral treatment.

In conclusion, we have reported the process and protocol that arose from the development of a ketamine treatment pathway for TRD. We believe this protocol is of interest to services and psychiatrists wishing to respond increasing demand for ketamine treatment. We believe the protocol provides a practical pathway that could be replicated in other services, and lead to greater availability of ketamine for patients with TRD.

## Supporting information

Beaglehole et al. supplementary material 1 Beaglehole et al. supplementary material

Beaglehole et al. supplementary material 2 Beaglehole et al. supplementary material

## Data Availability

Data availability is not applicable to this article as no new data were created or analysed in this study.
