# Supplementary material for: Multidisciplinary development of guidelines for ketamine treatment for treatment-resistant major depression disorder for use by adult specialist mental health services in New Zealand
Source: BJPsych Open. 2023 Oct 13;9(6):e191. doi: 10.1192/bjo.2023.577 (PMC10594164; doi:10.1192/bjo.2023.577)
Supplement: Beaglehole et al. supplementary material 1 — Beaglehole et al. supplementary material [file S205647242300577Xsup001.docx]

Ketamine Treatment Audit Form

| Patient name: | DOB: |
| --- | --- |
| NHI: | Treating team: |
| Psychiatrist: | Case manager: |
| Informed consent received | Baseline measures completed 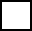 |
| Planned first treatment date: | Proposed treatment pathway (IM or oral): |
| Indication for treatment: | |
| Psychiatric comorbidities: | |
| Medical comorbidities: | |
| Current medications: | |
| Allergies: | |
| Referrers Name: Signature: Date: | |

# Treatment Record

| Treatment | Date | Dose (mg/kg) | Total Dose | Comments |
| --- | --- | --- | --- | --- |
| 1 |  |  |  |  |
| 2 |  |  |  |  |
| 3 |  |  |  |  |
| 4 |  |  |  |  |
| 5 |  |  |  |  |
| 6 |  |  |  |  |
| 7 |  |  |  |  |
| 8 |  |  |  |  |
| 9 |  |  |  |  |
| 10 |  |  |  |  |
| 11 |  |  |  |  |
| 12 |  |  |  |  |
| 13 |  |  |  |  |
| 14 |  |  |  |  |
| 15 |  |  |  |  |
| 16 |  |  |  |  |
| 17 |  |  |  |  |
| 18 |  |  |  |  |
| 19 |  |  |  |  |
| 20 |  |  |  |  |
| 21 |  |  |  |  |
| 22 |  |  |  |  |
| 23 |  |  |  |  |
| 24 |  |  |  |  |
| 25 |  |  |  |  |

# Name: __________________ NHI: _________________

# DASS-21 Scores

|  | Date | Score |
| --- | --- | --- |
| Baseline |  |  |
| Week 1 |  |  |
| Week 2 |  |  |
| Week 3 |  |  |
| Week 4 |  |  |
| Week 5 |  |  |
| Week 6 |  |  |
| Week 7 |  |  |
| Week 8 |  |  |
| Week 9 |  |  |
| Week 10 |  |  |
| Week 11 |  |  |
| Week 12 |  |  |
| Post treatment |  |  |

# Bladder Symptoms

| Baseline |  |
| --- | --- |
| Week 4 |  |
| Week 8 |  |
| Week 12 |  |
| Post treatment |  |

Memory and other side effects and date reported:

**DASS21** Name: Date:

DASS-21

Please read each statement and circle a number 0, 1, 2 or 3 which indicates how much the statement applied to you *over the past week.* There are no right or wrong answers. Do not spend too much time on any statement.

*The rating scale is as follows:*

1. Did not apply to me at all
2. Applied to me to some degree, or some of the time
3. Applied to me to a considerable degree, or a good part of time
4. Applied to me very much, or most of the time

| 1 | I found it hard to wind down |  | 0 | 1 | 2 | 3 |
| --- | --- | --- | --- | --- | --- | --- |
| 2 | I was aware of dryness of my mouth |  | 0 | 1 | 2 | 3 |
| 3 | I couldn’t seem to experience any positive feeling at all |  | 0 | 1 | 2 | 3 |
| 4 | I experienced breathing difficulty (eg excessively rapid breathing, breathlessness in the absence of physical exertion) |  | 0 | 1 | 2 | 3 |
| 5 | I found it difficult to work up the initiative to do things |  | 0 | 1 | 2 | 3 |
| 6 | I tended to over-react to situations |  | 0 | 1 | 2 | 3 |
| 7 | I experienced trembling (eg in the hands) |  | 0 | 1 | 2 | 3 |
| 8 | I felt that I was using a lot of nervous energy |  | 0 | 1 | 2 | 3 |
| 9 | I was worried about situations in which I might panic and make a fool of myself |  | 0 | 1 | 2 | 3 |
| 10 | I felt that I had nothing to look forward to |  | 0 | 1 | 2 | 3 |
| 11 | I found myself getting agitated |  | 0 | 1 | 2 | 3 |
| 12 | I found it difficult to relax |  | 0 | 1 | 2 | 3 |
| 13 | I felt down-hearted and blue |  | 0 | 1 | 2 | 3 |
| 14 | I was intolerant of anything that kept me from getting on with what I was doing |  | 0 | 1 | 2 | 3 |
| 15 | I felt I was close to panic |  | 0 | 1 | 2 | 3 |
| 16 | I was unable to become enthusiastic about anything |  | 0 | 1 | 2 | 3 |
| 17 | I felt I wasn’t worth much as a person |  | 0 | 1 | 2 | 3 |
| 18 | I felt that I was rather touchy |  | 0 | 1 | 2 | 3 |
| 19 | I was aware of the action of my heart in the absence of physical exertion (eg sense of heart rate increase, heart missing a beat) |  | 0 | 1 | 2 | 3 |
| 20 | I felt scared without any good reason |  | 0 | 1 | 2 | 3 |
| 21 | I felt that life was meaningless |  | 0 | 1 | 2 | 3 |

Total: __________________

**Questions regarding Bladder Pain or Interstitial Cystitis:**

1. Have you experienced an increase in frequency of urination or increased urgency to pass urine since commencing ketamine treatment?
2. Have you experienced new pain in the bladder or pelvic region since commencing ketamine treatment?
3. Have you noticed any blood in your urine?

**Screening questions for other side effects:**

1. Have you had any concerns with your memory or other thought processes since commencing ketamine treatment?
2. Are there any new physical or mental health concerns that you have since commencing ketamine treatment?
