# Supplementary material for: Multidisciplinary development of guidelines for ketamine treatment for treatment-resistant major depression disorder for use by adult specialist mental health services in New Zealand
Source: BJPsych Open. 2023 Oct 13;9(6):e191. doi: 10.1192/bjo.2023.577 (PMC10594164; doi:10.1192/bjo.2023.577)
Supplement: Beaglehole et al. supplementary material 2 — Beaglehole et al. supplementary material [file S205647242300577Xsup002.docx]

**Guidance for off-label ketamine use for Treatment Resistant Major Depressive Disorder**

**Authors:** Dr Ben Beaglehole, Dr Mike Clarke, Prof Richard Porter, Prof Paul Glue.

**Consultation group:**

- Clinical Leadership
- Clinical Pharmacy
- Academic
- Electro-Convulsive Therapy (ECT) service
- Service user
- Nursing
- Korowai Atawhai (the Māori mental health service)

**Background**

The short-term efficacy for racemic ketamine treatment of Treatment Resistant Major Depressive Disorder (TRD) is increasingly well-established. However, there are challenges for the translation of this research base into routine care. These include:

- The predominant use of parenteral modes of administration (IV, SC, IM)
- Marked dissociative effects with parenteral administration
- High rates of relapse following single or short-term repeated dosing
- Concerns about the potential for dependence and misuse.
- High rates of bladder/interstitial cystitis amongst high dose/high frequency recreational ketamine users
- Cognitive/memory impairment amongst high dose/high frequency recreational ketamine users
- Lack of ketamine treatment expertise amongst SMHS staff

The Royal Australian and New Zealand College of Psychiatry (RANZCP) has published a clinical memorandum to provide information to psychiatrists about the potential utility of ketamine in clinical practise^1^. This memorandum does not provide sufficient practical advice for psychiatrists wishing to commence treatment but is a useful benchmark for services to consider and ensure ketamine treatment is appropriate.

**Esketamine**

In NZ, Esketamine (the S-enantiomer of Ketamine) is available for TRD in combination with a conventional antidepressant and is delivered via nasal spray. Esketamine is not funded by Pharmac (and is therefore costly for consumers) and can only be administered during a 2-3 hour visit to an appropriate clinic, making it an expensive treatment for health services. The RANZCP mood disorder guidelines also report caveats that Esketamine has not been compared directly with Ketamine, the majority of Esketamine data stems from industry-sponsored trials, and that longer-term outcomes with this formulation are still a matter for debate^2^.

**Ketamine injection**

Ketamine injection is utilised in various forms of medicine including pre-ECT, emergency sedation and pre-surgery. Ketamine injection is funded for the above indications but is not funded or licensed for TRD in New Zealand. There is no readily available oral tablet formulation of ketamine but ketamine injection can be administered orally, IM or IV. Oral ketamine is subject to first pass metabolism. There is an oral bioavailability of approximately 30% with maximum concentrations reached after 30 minutes. It has an unpleasant taste so is generally mixed with a strong flavoured liquid. Common adverse effects of oral ketamine include nausea, vomiting, blurred vision, dizziness and headache. Ketamine is metabolised by CYP450 enzymes 2B6 and 3A4 enzymes and drugs that inhibit or induce these enzymes may increase or decrease the concentrations of ketamine. Coadministration with other sedating medication requires caution but other interactions appear minimal. A small case series has also reported on the concurrent use of ketamine with Monoamine Oxidase Inhibitors^3^. Although significant interactions were not present, the small number of cases suggests caution should still be undertaken with this combination of medication.

**Aim**

To create ketamine treatment guidelines for a publicly funded Specialist Mental Health Service (SMHS) through a collaborative process involving SMHS leadership, pharmacy, academic, ECT, service user, and nursing representatives. A secondary aim is to create a useful protocol for other publicly funded SMHS and publish this protocol for wider dissemination.

**Primary indication**

The primary patient population will be adult patients with TRD. Given the challenges associated with ketamine treatment, we propose restricting ketamine treatment to those with TRD of a severity to require treatment by SMHS. Proposed patients will therefore be receiving treatment for TRD from Adult Community Psychiatric Services and be allocated a case manager and overseeing psychiatrist. We expect TRD patients to have trialled at least two antidepressant treatments (of adequate dose and duration) and evidence-based psychotherapy for MDD (if available).

**Exclusion criteria**

- Inability to consent for treatment
- Significant comorbid Personality Disorder
- Active substance use disorder
- Primary Psychotic Disorder
- Dementia
- Significant Bladder pathology
- Allergy to ketamine
- Significant Hypertension/Cardio-vascular disease (for the Rapid test of ketamine response pathway only)
- Other significant acute or chronic medical comorbidity
- Failed course of ketamine in the past 12-months

**Ketamine treatment**

We include two ketamine treatment options. Clinicians and patients to select one of the following:

1. *Rapid test of ketamine response pathway*

- If determining whether patient is responsive to ketamine is urgent, patients can be given 1mg/kg IM ketamine at the clinic of the local psychiatric hospital (where medical monitoring and support is available).
- If this pathway is chosen, the case manager and psychiatrist will both attend for ketamine dosing and remain until the acute dissociative reaction has subsided. Close supports/whanau may also attend. If the psychiatrist has observed <4 ketamine IM administrations, a medical practitioner experienced in ketamine treatment is required to support treatment administration.
- Pre-medication will include ondansetron 4mg orally 30min pre-dosing.
- Ketamine monitoring will include baseline Blood Pressure (BP), SaO2 (oxygen saturations), HR (heart rate). Ketamine not to proceed if BP>140/90 at baseline or HR>100/min. BP, HR at 15-minute intervals until returned to baseline.
- Patients should expect to be at the Clinical Services Unit (CSU) for 3 hours including two hours post-dose. Patients need to be oriented, alert, mobile, and with stable observations prior to departure.
- Self-report Depression, Anxiety and Stress scale (DASS-21)^4^ to be completed at baseline and 48 hours post-treatment to check if ketamine responsive and whether to continue with oral ketamine pathway.
- Repeated IM injections are outside of the pathway. If patient is ketamine responsive, they will have the option of continuing with the Standard oral ketamine pathway outlined next.

1. *Standard oral Ketamine pathway*

- Overseeing psychiatrist to prescribe ketamine according to protocol and discuss with a medical practitioner experienced in ketamine treatment if needed. Case manager and/or team nurse to administer oral ketamine in an appropriate setting (home or clinic) and provide 1:1 support during first dose. Supports/whanau welcome to attend.
- Standard racemic ketamine for injection to be used mixed with 100mls orange juice and sipped over 30-60 minutes. Oral dosing and sipping over 30+ minutes will prevent significant dissociation and sympathetic response, by minimizing peak drug concentrations.
- Initial dose 1mg/kg. According to DASS-21 and clinical response, ketamine can be increased to 1.5mg/kg and then 2mg/kg if response is suboptimal.
- Dose frequency is once or twice weekly depending on duration of response. Exceptionally this could be three times/week, but only for patients where there is a clear improvement post dosing, and a rapid return of depressive symptoms. The course of ketamine is up to 12-weeks duration. This duration was chosen to provide sufficient opportunity for a robust, sustained clinical response with opportunity for behavioural change to increase the likelihood of a more enduring improvement in wellbeing.

**Informed consent**

Informed consent is required to proceed with ketamine treatment. This process will be completed by the treating psychiatrist. The decision-making process will be documented using a written consent form to ensure the following are discussed:

- That ketamine is being used off label.
- The risks and benefits of ketamine treatment, and alternative treatments.
- The dissociative reaction associated with IM ketamine is explained if this pathway is chosen
- The short-term nature of ketamine response, and the high risk of relapse following the course of treatment ending
- The potential for dependence and misuse.
- Ketamine bladder/interstitial cystitis amongst high dose and high frequency recreational ketamine users
- Cognitive/memory impairment amongst high dose and high frequency recreational ketamine users
- The monitoring requirements for participation in the ketamine clinic.

**Monitoring**

- Mood: Patients will be monitored clinically by their case managers on at least a weekly basis during ketamine treatment. Ketamine treatment monitoring will be supplemented by the DASS-21 ^4^ at baseline and at least weekly intervals during ketamine treatment. A further DASS-21 will be completed six weeks following treatment ended. The baseline and weekly DASS-21 scores will be collected by the patient’s case manager and prompt regular discussion with the psychiatrist about the clinical status of the patient including any risk issues and the adequacy of ketamine dose. Psychiatrist review should occur the week following the IM ketamine dose (if used) or 2-weeks after ketamine initiation and then occur as needed.
- Cognition: In most cases this will be monitored simply by enquiry of the patient and family/whanau if available. More formal testing will be considered if there are complaints of cognitive difficulties.
- Screening for urinary tract symptoms will be undertaken pre-ketamine treatment and monitored monthly during the course of treatment. Suggested screening questions are provided in the Supplement A.

**Audit**

Demographic characteristics and the DASS-21 results will be collated and reported to the steering group every three months. The steering group will meet three-monthly until confident the ketamine treatment guidelines and ketamine treatment are working satisfactorily. The data will be collated by the case manager. We considered using the Ketamine Side Effect Tool (KSET) ^5^ to monitor side effects but chose not to do so because of the complexity of this tool. Supplement A is our suggested form to document ketamine treatment and side effects.

**Service requirements**

A nominated service is required to collate data for audit purposes.

IM doses of ketamine will be administered at the ECT department (where medical monitoring is available) or in inpatient facilities.

Oral ketamine can be supervised in other settings including general outpatient services and patients’ homes.

The responsibilities for patients receiving ketamine treatment and collecting data for audit are with their treating teams.

**Driving**

Patients will be advised not to drive or operate heavy machinery on the day of IM or oral ketamine dosing. Taxis or the use of support to provide pickup are needed for transport after IM ketamine dosing.

**Adjunctive treatment**

Patients will continue their usual medication regimes. Given the short-term benefits of ketamine treatment, consideration of treatment changes to confer longer-term benefits should be considered. Adjunctive psychotherapy and active input from case managers are recommended during ketamine treatment to reinforce any mood improvements with behavioural change.

**Non-TRD patients**

TRD patients will be prioritised while the ketamine clinic is being established and ketamine expertise is growing. Following establishment of the clinic, patients with severe treatment resistant PTSD and other anxiety disorders may be considered if the evidence base continues to grow in this area.

**Repeat courses and maintenance treatment**

Community rates of TRD are significant and associated with individual, family, and societal morbidity. However, SMHS resources are limited and treatment is restricted to those with most severe TRD. Unless there are exceptional circumstances, the course duration will be 12 weeks with repeat courses available annually. As clarity around demand emerges, maintenance treatment will be considered on a case-by-case basis although longer-term evidence of ketamine is limited.

**ECT**

ECT is the gold-standard intervention for TRD; particularly if there is psychotic features, severe melancholia or very high risks to self resulting from self-neglect or suicide. Patients with TRD of this severity typically require inpatient treatment and are unlikely to be represented in clinical trials of ketamine treatment. In this context, ketamine treatment is better regarded as an alternative to ECT for less severe TRD or preceding ECT in a TRD algorithm.

**Special populations**

This protocol was designed to provide a ketamine treatment pathway for adults with TRD. We did not consult with specialists working in sub-specialty areas such as maternal and infant psychiatry, old-age psychiatry, and child and adolescent psychiatry. If ketamine treatment is considered for patients in these areas, we suggest consideration is given to a clinical pharmacy opinion (in cases of medical comorbidity and polypharmacy) and a psychiatric second opinion.

**Internal benchmarking against RANZCP clinical memorandum on use of ketamine (July 2022) key messages^1^**

- Ketamine is a complex drug that has strong effects on the mental and physical states of patients. Psychiatrists should ensure they are fully aware of these effects and required precautions when treating patients with ketamine. *Agreed and consistent with suggested protocol.*
- Ketamine is emerging as a new treatment in psychiatry, but further active research is required to understand how to optimally use ketamine for treating mental illness. *Agreed; a cautious approach endorsed by SMHS is required.*
- At present there is sufficient evidence only for use in treatment-resistant depression, and not any other psychiatric conditions. Ketamine is not recommended as a first line treatment and should only be initiated after due consideration of published evidence for its use. *Agreed; our protocol is primarily for TRD. However, the evidence base is evolving and non-TRD indications may emerge and be considered with appropriate consultation and over time.*
- Ketamine treatment should only be initiated after assessment by a psychiatrist familiar with the evidence and effects of ketamine. Only a psychiatrist or a medical practitioner (under the supervision of a psychiatrist) with appropriate expertise in ketamine treatment should prescribe ketamine and take responsibility for its use in treating depression. *Partial agreement; we are primarily using oral ketamine which is more straightforward to prescribe and the purpose of providing a protocol with layers of support is to ensure expertise in ketamine treatment is more widely available as opposed to restricted to a small number of practitioners.*
- Ongoing research into ketamine under research trial conditions is encouraged, including oversight by an institutional research ethics committee and careful monitoring and reporting of effectiveness and safety outcomes. *Agreed. We plan to submit this ketamine protocol for peer review and publication and collect data for audit, monitoring, and improvement.*
- Psychiatrists who are considering prescribing ketamine for a patient outside of a research trial should ensure they are fully familiar with the complexities of ketamine dosing and management and have established appropriate clinical infrastructure for such treatment. This includes an appropriate structured framework for safety monitoring and outcome measures for the recording of side effects and benefits when used in clinical practice. *Agreed; embedded within the protocol.*
- Services providing ketamine therapy should have clear practice policies and guidelines for its use. *Agreed; embedded within the protocol.*

Permission has been granted by the RANZCP for the use of these key messages.

**Suggested Patient Information Sheet: Ketamine treatment for Treatment-Resistant Depression**

**What is Treatment-Resistant Depression (TRD)?**

Depression is the most common mental illness in the community. Depression is characterised by feeling low in mood and not being able to enjoy life. Depression is usually accompanied by over or under-sleeping, appetite changes, energy changes, and impaired concentration. Thinking usually becomes negative and may include suicidal thoughts.

TRD is diagnosed when depression persists despite trying antidepressants and psychotherapy (talk therapy).

**What is ketamine?**

Ketamine is a medication used in the management of severe pain and sometimes anaesthesia. For the past decade or more, clinicians have studied ketamine for the treatment of mental illnesses.

**Ketamine treatment for TRD**

Ketamine is an effective short-term treatment for TRD although not everybody tolerates ketamine or responds to treatment. The majority of studies have investigated ketamine injections but oral ketamine (ketamine mixed with orange juice and sipped over 30-60 minutes) has also been used and appears beneficial.

Ketamine use is off-label in New Zealand although Esketamine (closely related to ketamine) is registered for TRD. Esketamine is not funded and is therefore expensive for consumers.

**Side effects of ketamine**

Ketamine injections are associated with a severe ‘dissociative’ experience. Patients feel disconnected from their usual senses and time for approximately 45 minutes. They may become drowsy or immersed in past memories. Nausea is common so we provide an anti-nausea medication (ondansetron) before the injection.

Oral ketamine is usually better tolerated with minimal dissociation or nausea.

Ketamine causes short term increases in blood pressure and heart rate that subside as the effects wear off.

Ketamine is a recreational drug used by some people. It can be associated with dependence or addiction.

Frequent, high dose use of ketamine by drug users is associated with bladder symptoms (difficulty passing urine) and memory impairment (forgetfulness) but these concerns have not been evident when low-dose ketamine has been used for psychiatric reasons.

**Important things to consider**

We offer a single intramuscular injection of ketamine as a rapid way of determining whether you are likely to respond to oral ketamine. The dissociative effects of ketamine can be scary although many people manage the experience without much difficulty. If you are responsive to ketamine, you will be offered a course of oral ketamine to follow.

If you take regular medications, these will not change and you are advised to continue taking them as prescribed throughout Ketamine treatment.

If you do not wish to have the injection you can still receive the course of oral ketamine. We anticipate this will be more suitable for most people as it avoids the injection and the severe dissociative experience. There may be some situations such as severe suicidal thoughts where your psychiatrist may recommend starting with the injection as a better option.

Ketamine is only associated with short-term benefits. Typically, mood feels better for several days before deteriorating again. Repeated doses offer the possibility of more sustained improvement during the course of treatment.

The risk of depression relapse is still high after single or repeated doses. We offer ketamine treatment as a 12-week course. We believe this time frame provides a good opportunity for better control of symptoms. We believe the course of treatment also offers you the chance to make changes that may prevent relapse after ketamine stops.

It is important that you discuss ketamine treatment with your family/whānau and your treating team before deciding if you want to proceed with ketamine treatment.

**Patient flowchart**

Discussion with your doctor and whanau about whether you would like to proceed with Ketamine treatment

**Yes**

**No**

Your doctor/nurse will take measurements such as your blood pressure and heart rate, medical history to make sure Ketamine is a safe treatment for you.

Other treatment options will be discussed

If you respond to the Ketamine injection you may continue with Oral Ketamine treatment

Do you wish to have the **Rapid Ketamine Injection?**

This will determine if you are likely to respond to Ketamine treatment

You will be given Oral Ketamine ONCE or TWICE weekly for a maximum of 12 weeks (3 months).

**Yes**

**No**

Your doctor will discuss if this is appropriate for you

If your doctor thinks it is appropriate: You will be given an anti-nausea medicine then an injection of Ketamine into your upper arm at the Mental Health Clinic. You will feel disassociated (out of it) for about 45 minutes and you will be at the unit for around 3 hours. Your nurse/doctor will look after you during this time.

It is important you have a friend/family member to pick you up after treatment.

**No**

**Yes**

If you respond to the Ketamine injection you may choose to continue with Oral Ketamine treatment

If your doctor thinks it is appropriate: You will proceed with **Oral Ketamine treatment** (ketamine mixed with orange juice)

Your nurse/case manager will give you oral Ketamine in your home or at the outpatient clinic to sip over 30-60 minutes. This will be a milder experience than what you may have experienced with the Ketamine injection. This will take approximately 60 minutes.

It is important you have a friend/family member to pick you up after treatment.

Your doctor and nurse will follow-up with you to decide further treatment

Your doctor and nurse will follow-up with you to decide further treatment

**Suggested consent form for off-label ketamine treatment of depression**

This form is to be completed following a discussion about ketamine treatment and its alternatives with the consultant psychiatrist overseeing care. It is important to take the time to make a good decision and discuss ketamine treatment with family/whanau and other supports.

I, …………………………………………………………………………………….., consent to receiving treatment with one of the following (please delete the non-applicable option):

- *Rapid test of ketamine response option (Intramuscular injection of ketamine. If ketamine responsive, a course of oral ketamine once or twice weekly for a maximum of 12 consecutive weeks will be offered)*
- *Standard oral ketamine pathway (oral ketamine once or twice weekly for a maximum of 12 consecutive weeks).*

In giving consent, I acknowledge that I have discussed the pros and cons of ketamine treatment including alternative options with my psychiatrist. This discussion has included the following:

- The intense dissociative reaction (spacing out) experience with Intramuscular ketamine. Dissociative side effects of intramuscular ketamine include time speeding or slowing, colours changing in intensity, reliving old memories and altered sensations (delete if not applicable)
- The risk of high blood pressure with Intramuscular ketamine (delete if not applicable)
- Oral ketamine is associated with temporary mild effects including disinhibition and euphoria (extreme happiness)
- The short-term clinical response to ketamine
- The risk of relapse following a course of ketamine treatment
- The risk of bladder symptoms following ketamine treatment (this is largely an issue with high dose frequent recreational users)
- The risk of memory problems with ketamine treatment (this is largely an issue with high dose frequent recreational users)
- The risks of recreational/misuse of ketamine

Signed:

………………………………………………………………………………………………..date……………………………………………..

Psychiatrist:

Name……………………………………………………..Signature…………………………………………………..date……………

References

1. Committee for Evidence-Based Practice. Use of ketamine in psychiatric practice. Royal Australian and New Zealand College of Psychiatry (RANZCP), 2022.

2. Malhi GS, Bell E, Bassett D, Boyce P, Bryant R, Hazell P, et al. The 2020 Royal Australian and New Zealand College of Psychiatrists clinical practice guidelines for mood disorders. Aust N Z J Psychiatry. 2021; 55(1): 7-117.

3. Katz RB, Toprak M, Wilkinson ST, Sanacora G, Ostroff R. Concurrent use of ketamine and monoamine oxidase inhibitors in the treatment of depression: A letter to the editor. Gen Hosp Psychiatry. 2018; 54: 62-4.

4. Lovibond SH, Lovibond PF. Manual for the Depression Anxiety Stress Scales. Psychology Foundation, 1995.

5. Short B, Dong V, Galvez V, Vulovic V, Martin D, Bayes AJ, et al. Development of the Ketamine Side Effect Tool (KSET). J Affect Disord. 2020; 266: 615-20.
